# Tunicates: exploring the sea shores and roaming the open ocean. A tribute to Thomas Huxley

**DOI:** 10.1098/rsob.150053

**Published:** 2015-06-17

**Authors:** Patrick Lemaire, Jacques Piette

**Affiliations:** Centre de Recherches de Biochimie Macromoléculaire. UMR 5237, Centre National de la Recherche Scientifique, Université de Montpellier, 1919 Route de Mende, 34293, Montpellier cedex 5, France

**Keywords:** tunicates, thaliaceans, appendicularians

## Abstract

This review is a tribute to the remarkable contributions of Thomas Huxley to the biology of tunicates, the likely sister group of vertebrates. In 1851, the great biologist and philosopher published two landmark papers on pelagic tunicates in the *Philosophical Transactions of the Royal Society*. They were dedicated to the description of the adult anatomy and life cycle of thaliaceans and appendicularians, the pelagic relatives of ascidians. In the first part of this review, we discuss the novel anatomical observations and evolutionary hypotheses made by Huxley, which would have a lasting influence on tunicate biology. We also briefly comment on the more philosophical reflections of Huxley on individuality. In the second part, we stress the originality and relevance of past and future studies of tunicates in the resolution of major biological issues. In particular, we focus on the complex relationship between genotype and phenotype and the phenomenon of developmental system drift. We propose that more than 150 years after Huxley's papers, tunicate embryos are still worth studying in their own right, independently of their evolutionary proximity to vertebrates, as they provide original and crucial insights into the process of animal evolution. Tunicates are still at the forefront of biological research.

## 1. Introduction

In 1851, two landmark articles by British zoologist Thomas Huxley were published side by side in the *Philosophical Transactions of the Royal Society* [1,2]. These pieces of work, one of which is reproduced in this issue, were dedicated to the description of the adult anatomy and life cycle of some of the most enigmatic pelagic invertebrates: the salps, pyrosomes and doliolids, now composing the thaliaceans, and the appendicularians. Huxley proposed for the first time that, similarly to salps [3], appendicularians are closely related to ascidians, and thus belong to the tunicates, now considered to be the sister group of vertebrates. He further suggested that they are the ‘lowest’ form of tunicates. His careful description of the complex life cycle of salps also led him to challenge the classical notion of ‘individuality’.

**Figure 1.**
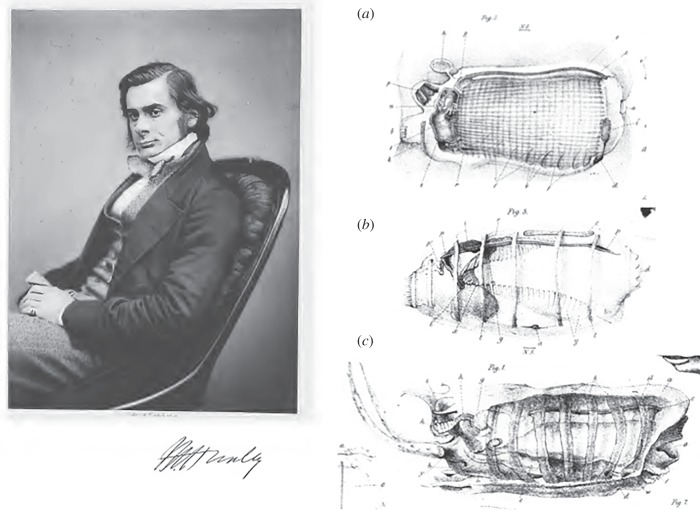
Portrait of T. H. Huxley and representative drawings of a zooid of *Pyrosoma* (*a*), a blastozooid of *Doliolum denticulatum* (*b*) and an oozooid of *Thalia democratica* (*c*). Note that, as Huxley was not aware of the close relationship between tunicates and vertebrates, the embryos are shown in reverse dorsoventral orientation with the endostyle upwards and the brain downwards. (*a*,*c*) Adapted from [2] and (*b*) from [1].

Huxley's papers are characterized by much detailed and accurate anatomical descriptions and are beautifully illustrated (figure 1). They still provide a particularly rewarding read, for the accuracy and conceptual importance of their scientific content as well as for their style. Indeed, the modern scientific reader is seldom confronted with poetic evocations of the mysteries of the sea: ‘The sky was clear but moonless, and the sea calm; and a more beautiful sight can hardly be imagined than that presented from the decks of the ship as she drifted, hour after hour, through this shoal of miniature pillars of fire gleaming out of the dark sea, with an ever waning, ever-brightening, soft bluish light, as far as the eye could reach on every side’ [1, p. 580].

Here, we will present a brief overview of the contribution of Thomas Huxley to the biology of tunicates and highlight some of the current biological and evolutionary riddles that these fascinating creatures can help elucidate.

## 2. Classifying nature and the origin of species: tunicate phylogeny, then and now

A short presentation of the tunicates is given in box 1. The sessile ascidians (or sea squirts), represented by *Ciona intestinalis*, are the best-known representatives of this group, for which the status of phylum was recently proposed [4]. The close relationship of the pelagic salps with the ascidians was first inferred by Cuvier in 1804 [3]. Lamarck clearly differentiated these marine animals from molluscs, into which they were initially classified, coining the term ‘tuniciers’ (tunicates) because of the double tunic encasing and protecting the adult [5], and formed in part by cellulose material as later shown by Huxley [6]. The pyrosomes were recognized to belong to the tunicates in the first half of the nineteenth century [7] and the doliolids were subsequently added to the group by Quoy and Gaymard, in their report of their journey on the *Astrolabe* [8]. By the time Huxley boarded the HMS *Rattlesnake* in 1846 as an assistant surgeon, the tunicates were composed of ascidians, salps, doliolids and pyrosomes but the few available descriptions of the latter three clades remained rare and superficial, and their life history remained enigmatic, with the notable exception of Chamisso's description of the alternation of generations in salps [9]. Appendicularians had previously been described by Chamisso [10], Mertens [11] and Quoy & Gaimard [12] who had not recognized the similitude to the other tunicates.

Box 1. **Tunicates.**

Tunicates are composed of three main groups, the sessile ascidians on one hand and the pelagic appendicularians and thaliaceans on the other. They are characterized by the possession of a tunic composed of cellulose. Adult tunicates are filter feeders: the seawater enters a pharynx through an inhalating or oral siphon, in most cases set in motion by ciliary beating, food particles are trapped on a mucous net secreted by the endostyle, and the water and waste exit the body through an exhalating or atrial siphon. Appendicularia, ascidians and some thaliaceans possess a tadpole-like larva with a notochord and metamorphose into sessile adults in the case of ascidians. Tunicates have reversible blood flow. See text for further details.
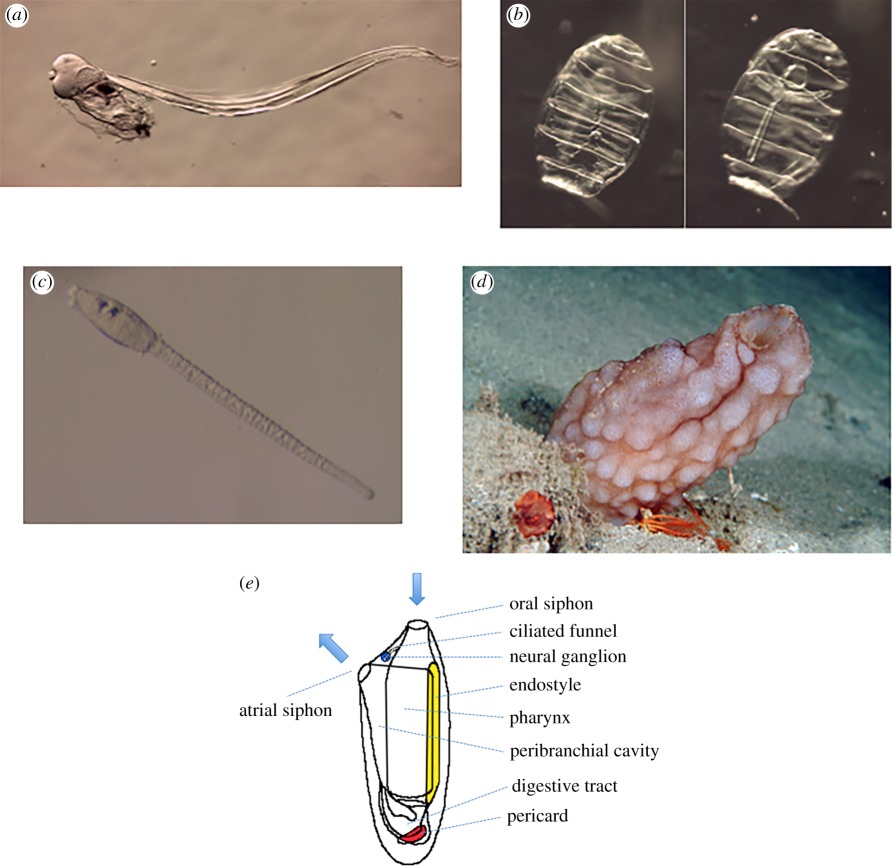
Tunicates are composed of three groups. (*a*) Live images of an adult Appendicularia, (*b*) two focal planes (left, dorsal with the neural ganglion, right, ventral with the endostyle, note the muscle bands encircling the zooid) of a blastozoid of the thaliacean *Doliolum nationalis*, (*c*) a larva of the ascidian *Phallusia mammillata* and (*d*) an adult of the same species, (*e*) Bauplan of an adult ascidian (modified after Brien, 1946).

In his two 1851 articles, written shortly after the return of the *Rattlesnake*, Huxley provides exquisitely detailed descriptions of the adult anatomy of salps and pyrosomes [1], and of appendicularians and doliolids [2], thereby shedding light on several tunicate organs and their functioning. He in particular described a novel organ, the endostyle, which he thought was a ‘very remarkable distinctive character of the Tunicata’ [1]. He also described the regular reversal of blood flow in salps [1], which he did not observe in doliolids [2]. The quality of his descriptions led to a refinement of the systematics and phylogeny of tunicates and rapidly entered one of the major monographs on the systematics of molluscs, ‘*Klassen und Ordnungen der Weichthiere*’ by Bronn [13]. The primacy of Huxley's descriptions was acknowledged by the German school, and in particular Leuckart [14] and Keferstein & Ehlers [15]. Krohn, unaware of Huxley's work, independently described the doliolids in 1852, missing the presence of the endostyle, in a piece of work translated into English, and criticized by Huxley, for the *Annals and Magazine of Natural History* [16].

**Figure 2.**
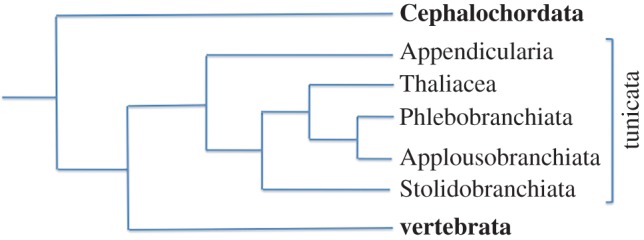
Phylogenetic tree reconstituted by a Bayesian approach avoiding long-branch attraction artefacts. Note that tunicates and vertebrates are sister groups as also the thaliaceans and phlebobranchs inside the ascidians.

Huxley first extended the tunicates by adding the Appendicularia to the group: ‘there can be no doubt that the animal is one of the Tunicata. The whole organization of the creature, its wide respiratory sac, its nervous system, its endostyle, all lead to this view’ [2, p. 599]. He also proposed evolutionary scenarios to explain tunicate diversity, based on the current evolutionary theories of his time. Influenced by the recapitulation theory, already suggested by the Greek philosopher Anaximander [17], formulated in its modern form by Meckel and Serres [18] and subsequently made popular by Haeckel in his biogenetic law (‘Ontogeny recapitulates Phylogeny’ [19]), he inferred the phylogenetic position of Appendicularia from the morphological comparison of their adult form with ascidian larvae: ‘as in all great natural groups some forms are found which typify, in their adult condition, the larval state of the higher forms of the group, so does Appendicularia typify, in its adult form, the larval state of the Ascidians’ [2, p. 599]. Although the biogenetic law has since been scientifically refuted, Huxley's conclusions on Appendicularia are supported by modern molecular phylogenies, which strongly suggest that this group is positioned at the root of the tunicate tree (figure 2) [20].

Huxley did not come to firm conclusions about the precise relationships between ascidians and thaliaceans. He challenged a previous classification of tunicates into Monochitonida and Dichitonida, based on the organization of their tunic, but did not propose an alternative [1]. Instead, he considered that the homology of inner structures among tunicates was such that it was difficult to ‘draw any very broad line of demarcation among the various members’ of the group, ‘the various genera passing one into the other by almost imperceptible gradations’ [1, p. 587], a suggestion in keeping with phyletic gradualism, already advocated by Leibniz and a major tenet of Darwin's theory.

Huxley made efforts to determine precise phylogenetic relationships between thaliaceans. He rejected the idea that salps and pyrosomes should only be distantly related [1]. Instead, he favoured the emergence of this group from a sessile colonial ascidian-like ancestor, because pyrosomes ‘have the closest similarity in structure to the Botryllidae and other compound Ascidians’ [1, p. 586] and considered the dorsal appendage of *Doliolum* as a rudiment of an ancestral pedicle of attachment [2]. In agreement with Huxley's proposal, thaliaceans appear monophyletic in current molecular phylogenies and are proposed to be nested within ascidians, where they form the sister group of the phlebobranchs [21] (first proposed by Wada [22]). Based on the structure of the branchial slits, and the position of the siphons and testis, Huxley proposed a pyrosoma–doliolum–salps evolutionary succession. Owing to the paucity of genomic resources for these taxa, their relative phylogenetic position remains controversial [23].

**Figure 3.**
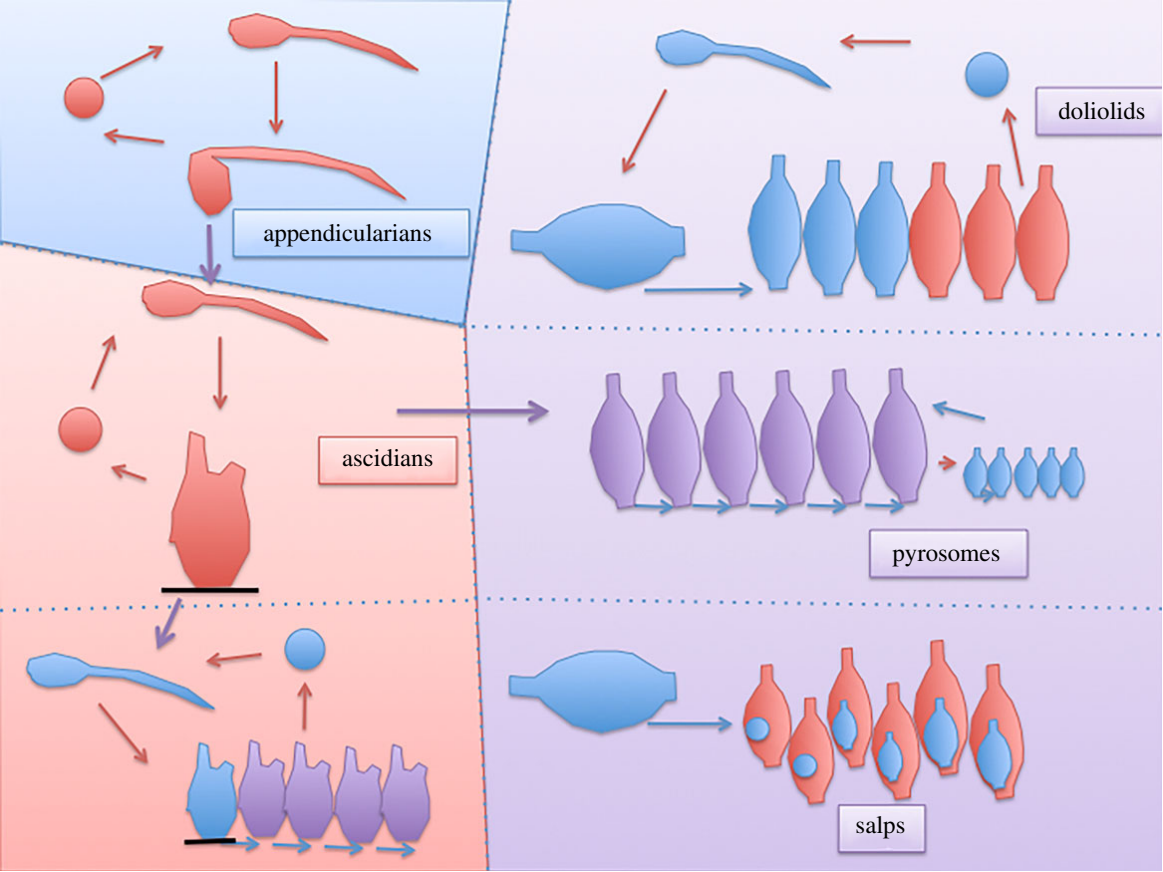
Evolutionary scenario of the tunicates. In this set-up, pelagic appendicularians gave rise to sessile ascidians, in which coloniality arose several times by budding, giving rise to applousobranchs and thaliaceans, the latter which returned to a pelagic life. Asexual generations are represented in blue, sexual in red, and mixed in purple.

Huxley did not specifically delve into the question of the sessile versus pelagic nature of the ancestral tunicates, a question which also remains controversial nowadays. There is a single convincing early tunicate fossil, *Shankouclava aningense* [24], a sessile ascidian-like creature from the Early Cambrian. Reconstitution of the ancestral tunicate therefore still relies in large part on phylogenetic analyses, followed by parsimony arguments. Most recent phylogenies consider appendicularians as the basal tunicate clade, suggesting that the last common ancestor (LCA) of the tunicates was pelagic [21] (F. Delsuc & E. Douzery 2015, personal communication), although this question is still debated [25,26]. According to this parsimonious scenario, sessility was acquired once by the LCA of ascidians and thaliaceans, and lost again in thaliaceans (figure 3). The latest molecular phylogeny thus argues for a free-swimming tunicate LCA.

Nature does not always follow parsimonious paths, however [27], and more complex scenarios cannot be formally excluded, including a sessile tunicate ancestor followed by a transition to pelagy in Appendicularia. Because metamorphosis is often considered diagnostic of a radical change in lifestyle between larval and adult forms [28], it is intriguing that appendicularians undergo metamorphosis, although both their adults and their larvae are pelagic. This could argue in favour of a sessile, or benthic, ancestor for tunicates (see also [29] for *Oikopleura*), in agreement with Garstang [30]. Finally, a pelagic ascidian ancestor, followed by the independent convergent acquisition of sessility in phlebobranchs and stolidobranchs, can also not be formally excluded, although evidence for a sessile ancestor for thaliaceans as proposed by Huxley has accumulated over time, and appears most likely [23].

## 3. Thaliaceans or how to radically change lifestyle and morphology

Our understanding of the ecology, development and life cycle of ascidians has much progressed since Huxley's times, suggesting scenarios for the emergence of pelagic thaliaceans from an ascidian-like ancestor.

Solitary ascidians show a remarkable conservation of embryonic cell lineages and morphologies and of larval development even between species that diverged a long time ago, such as the stolidobranch *Halocynthia roretzi* and the phlebobranch *C. intestinalis*. This stereotyped development is, however, not an evolutionary ‘dead-end’.

Indeed, colonial ascidian development departs from the solitary situation. There is an overall lengthening of embryogenesis, which can last up to five months [31], instead of 1 day in *Ciona* and *Halocynthia*. Unlike larvae of solitary ascidians, larvae of colonial ascidians are generally large, count many cells and hatch with well-differentiated adult structures [32]. In the majority of cases described, colonial species produce large eggs, and early development proceeds with the same early cell lineage as solitary species [33]. By contrast, some colonial aplousobranch species, such as *Hypsistozoa farmeriana* [31] have, like salps, a viviparous development during which embryos, produced from small eggs (25 µm in *H. farmeriana*), undergo considerable growth, and may strongly depart from the common solitary cell lineage. One can thus observe within ascidians a variety of both early and late developmental strategies.

Colonial ascidians illustrate that the formation of adult structures can thus become uncoupled from the necessity to adhere to a substrate observed in solitary ascidians. Interestingly, mutants that metamorphose without adhesion were recently identified in the solitary *Ciona*. These mutants are defective for the cellulose synthetase gene, and consequently produce celluloseless tunics [34]. The involvement of this type of mutant in the transition towards thaliacean pelagic life is unlikely, as cellulose is also found in the tunic of thaliaceans. Yet, the finding that a single mutation can alleviate the need for substrate adherence to undergo metamorphosis highlights that transition from sessility to pelagy needs not have involved extensive genetic rewiring in the LCA of ascidians and thaliaceans [35].

In addition to becoming pelagic, salps and pyrosomes have entirely lost the tadpole form, which was only retained in some doliolids [23]. Here, the thaliacean pelagic lifestyle may have alleviated the need for efficient larval dispersal, and thus the selective pressure for the maintenance of the swimming tadpole stage. Interestingly, loss of tailed tadpole stages has also been observed in several sessile Molgulidae and Styelidae ascidians [36]. In such cases, development is affected only at post-gastrula stages, and does not affect the final adult form. In the case of Molgulidae, it was proposed that the formation of anural larvae was driven by ‘positive selection for tadpole loss in highly adaptive habitats with patchy distribution […] because extensive dispersal may land the juvenile off the habitat and thus be lethal’ [37, p. 653]. Interestingly, tail loss may also involve a limited set of genetic changes, as a single main locus has been identified between two closely related tailed (*Molgula oculata*) and tailless (*Molgula occulta*) species [38].

## 4. The salp life cycle and a new definition of individuality

Thomas Huxley's work on salps led him to propose a novel definition of ‘zoological individuality’ (as opposed to ‘metaphysical individuality’), which he further developed in a separate monograph also published in 1851 [39]. This major conceptual advance took place at a time when studies of coloniality and asexual reproduction upset classical definitions of the individual (see for example Leuckart's paper, edited the same year, for the concept of division of labour between different members of the same colony [40]). While Huxley's anatomical discoveries were rapidly generally accepted, his more theoretical considerations were more controversial at the time.

Huxley's insight was based on his remarkable description of the life cycle of salps, which are found as both solitary and aggregate forms. Huxley's work is largely consistent with that of Chamisso [9], who had first understood that the solitary and aggregate forms of salps, initially classified as distinct species, represent alternating forms of the same species. Chamisso had introduced the concept of alternation of generations: he proposed that the solitary asexual form gives rise, by budding, to the aggregate sexual form, in which each ‘individual’ contains an embryo that will in turn develop into a solitary asexual form (figure 3). The alternation of generations was at the time a controversial topic, and the presence of ovocytes in the asexual, solitary oozoids led the American zoologist W. K. Brooks to object against alternation of generation in *Salpa* [41]. (See [23] for a more elaborate discussion of this topic.)

The exact definition of an ‘individual’ was the major point of disagreement between Huxley and Chamisso. For Huxley, the alternating forms are not to be considered as distinct individuals, but rather as successive organs of a single individual. In Huxley's words, an individual is ‘the sum of the phenomena successively manifested by, and proceeding from, a single ovum, whether these phenomena be invariably collocated in one point of space or distributed over many’ [1, p. 579]. Thus, an individual can be dispersed both in space and in time, in that it adopts radically different forms at different periods of its life, and sexual reproduction defines the boundaries of the individual. Huxley introduced the term ‘zooid’ to name the different forms of an individual, a term whose use has been extended to describe the life cycle of many phyla with colonial species. Interestingly, this close association between sexuality and individuality has been challenged by Huxley's own grandchild, Julian [42], who considered fertilization a poor boundary of individuality, as true twins are distinct individuals originating from a single fertilization event, and distinguished individuality in time from individuality in space (see [43] for an elaborate discussion of these topics).

## 5. Relationships between tunicates and vertebrates

Huxley's primary emphasis on the description of adult forms may have prevented him from recognizing that tunicates and vertebrates share embryonic structures that distinguish them from other animal groups, except cephalochordates, not studied by Huxley, and are thus phylogenetically closely related.

Although Huxley knew of the presence in Appendicularia of a central tail rod structure, he failed to make the connection with the embryonic notochord, a structure identified 25 years earlier by K. von Baer in early vertebrate embryos [44]. He thought the endostyle specific to tunicates, and did not recognize that vertebrates and cephalochordates possess homologous structures with divergent adult morphologies, including the vertebrate thyroid gland, and should thus be considered as chordate synapomorphies, shared derived traits characteristic of the group. The grouping of tunicates with vertebrates in a new phylum, the chordates, would have to wait until 1866 and the discovery by the Russian embryologist Alexander Kowalevski that the equivalent central rod in ascidian larvae is derived from a cellular structure similar to the vertebrate notochord [45].

**Figure 4.**
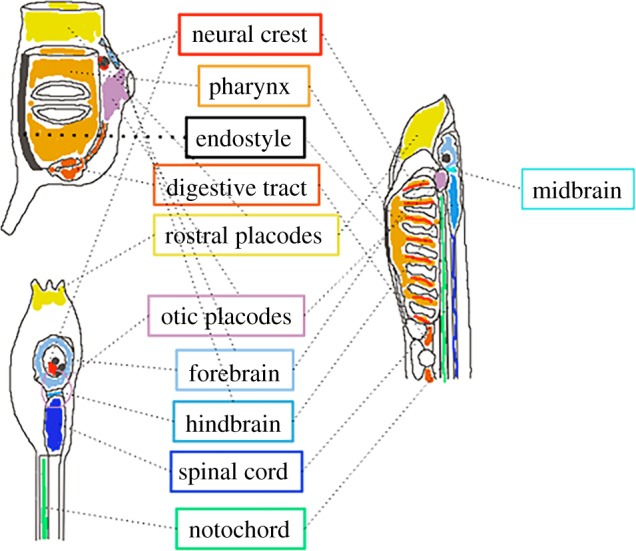
Possible homologies between tunicates and vertebrates. Larval (lower) and adult (upper) ascidians (*Ciona*) are represented at the left-hand side, while a vertebrate larva (lamprey) is represented at the right-hand side. Presumed homologous structures are boxed and schematized in similar colours.

Recently, improved Bayesian methods for the reconstruction of phylogenetic trees from large-scale sequencing projects have led to the unexpected conclusion that tunicates are the sister group of vertebrates [46] (figure 2). This molecular evidence contrasts with cladistic analyses [47], which placed cephalochordates as the most likely sister group to vertebrates because, in addition to the notochord and the dorsal neural tube shared by all chordates, they possess possible synapomorphies with vertebrates absent from tunicates, including segmented somites, and a postanal tail [47,48] (figure 4). More recently however, ascidians were shown to harbour synapomorphies with vertebrates not found in cephalochordates. For instance, the close association between the mouth (or oral siphon) primordium and anterior neural plate territories in both ascidians and amphibians suggests that the evolution of these two structures may be more closely linked than previously appreciated [49]. Also, ascidians and appendicularians develop sensory placodes, regions of thickened ectoderm that, in vertebrates, give rise to sensory neural structures [50,51]. One such ascidian structure, located anterior to the brain, most likely corresponds to the olfactory/adenohypophyseal placodes of vertebrates [50]. A pair of sensory placodes located on either side of the posterior brain bears most similarity to the vertebrate otic placodes fated to form the acoustico-lateralis system [51]. In ascidians, this structure forms the atrium and most of the atrial siphon of the adult. Also, *C. intestinalis* possesses a cephalic melanocyte lineage that has been proposed to be an evolutionary ‘precursor’ of the vertebrate neural crest [52]. Finally, analysis of the *Ciona* heart lineage suggests the presence of an ascidian evolutionary precursor of the vertebrate secondary heart field [53]. The longstanding difficulty in assigning an accurate phylogenetic position to tunicates within chordates may thus result from the combination of the loss in this group of some ancestral chordate plesiomorphies, including somite segmentation, with the emergence in olfactores of novel shared embryonic structures, which however give rise to adult organs with different morphologies, fulfilling distinct functions, and whose homology is thus difficult to recognize.

Divergence between tunicates and other chordates is also marked at the molecular level. Molecular phylogenies of tunicates, and in particular of *Oikopleura*, are characterized by long branches, reflecting rapid protein evolution. Gene loss is frequent, including genes that play fundamental roles in vertebrate development, such as *gbx2* [54] or the central *Hox* genes [55]. Fifteen per cent of *C. intestinalis* genes are organized in operons, a phenomenon associated to the presence of trans-splicing [56]. Non-coding sequences also evolve rapidly, and, while some ultra-conserved non-coding sequences are shared between vertebrates and cephalochordates [57], this is not the case with ascidians [58]. Although it has been reported that some sequences regulating gene expression in ascidian embryos are functional in vertebrates, closer analysis suggests that this could be artefactual [59]. Also, while cephalochordate genomes have retained substantial macrosynteny with vertebrates, *C. intestinalis* and *Oikopleura dioica* have not [60,61]. Finally, the *Hox* gene cluster of tunicates is dispersed over several chromosomes [55] and has a reduced role in the antero-posterior organization of the body plan compared with most bilateria [62].

In spite of this extensive divergence in genome sequence and architecture, orthologous regulatory genes sometimes play apparently conserved roles in homologous structures in ascidians and vertebrates (e.g. *Brachyury* for the notochord [47], *FGF8* for the midbrain–hindbrain boundary region [63], *Pax2/5/8*, *Six* genes and *Pitx* for placodes [51], *Mitf* in the pigmented cell lineage [52], and *Mesp* and *Gata4/5/6* in heart specification [64]). How conserved are the regulatory networks that act upstream or downstream of these shared regulators remains largely unknown. However, as considerable rewiring of genetic networks responsible for otherwise conserved traits (a phenomenon called developmental systems drift (DSD)) has occurred within tunicates [65,66], it is likely that differences between tunicates and vertebrates will be strong. For example, divergence in signalling strategies for motoneuron specification between ascidians and vertebrates were pinpointed [67,68]. Sobral and colleagues also reported that the expression profiles of orthologous ascidian and zebrafish genes, including regulatory genes, have considerably diverged [69]. This extensive molecular and anatomical divergence between tunicates and vertebrates suggests that extrapolations to vertebrates of results obtained with tunicates should only be done with caution.

## 6. Tunicates at the beginning of the twenty-first century: bridging DNA sequence and tissue morphogenesis

Tunicates entered the era of genomics and systems biology at the turn of the twenty-first century with the sequencing of the genomes of the phlebobranch ascidians *C. intestinalis* [54], and *Ciona savignyi* [70], followed by the appendicularian *O. dioica* [61] and the colonial stolidobranch ascidian *Botryllus schlosseri* [71]. Most of this work found its justification in the close phylogenetic relationship to vertebrates, and in the hope that the powerful experimental techniques developed for the simple ascidian embryos would shed light on the origins of vertebrates (figure 2).

*Ciona intestinalis* took the lead in this work. The compactness of its genome (approx. 150 Mb for approx. 15 000 coding genes) and the ease to perform and interpret *in situ* hybridization assays with cellular resolution led to high-resolution expression atlases of most regulatory genes acting up to the gastrula stage [63]. The development of electroporation methods to analyse *cis*-regulatory activity of defined sequences during early development [72], and the possibility to interfere with gene activity by microinjection of morpholinos [73], and more recently by expression of TALENs [74] or via CRISPR/Cas9 constructs [75], led to a detailed reconstruction of the early gene regulatory networks responsible for the establishment of the main tissue types of a chordate embryo [76]. This early gene regulatory network remains less complete than in sea urchins [77], and insufficient to systematically estimate its level of relatedness to vertebrate networks. Its further deciphering should, however, rapidly benefit from the recent improvements in single-cell transcriptomics, large-scale identification of enhancers based on the detection of open chromatin regions [78] and large-scale functional tests of these predicted enhancers [79]. Within the next few years, we anticipate to have a sufficiently complete understanding of the early *Ciona* gene regulatory networks to computationally simulate their dynamic functioning [80]. This wealth of information makes *C. intestinalis* the ‘golden standard’ for comparative studies with the developmental programmes of vertebrates and of other tunicates.

The optical transparency, cellular simplicity and stereotyped mode of development of ascidian and appendicularian embryos also provide a rigorous cellular framework in which the geometry and behaviour of individual cells can be imaged and reconstructed in three dimensions [81,82]. This approach, applied on a small scale, has revealed that cell signalling acts at very short range in early ascidian embryos [81], has provided a cellular basis for the observed narrowing of cells observed at each end of the notochord [83] and has allowed to dissect and mechanically simulate the first major morphogenetic event during ascidian embryogenesis, the invagination of the endodermal precursors [84]. Live quantitative imaging [85,86] and computational processing [87,88] of ascidian or appendicularian embryos will hopefully identify the repertoire of individual cell behaviours across development, and reveal how single cell behaviours integrate to produce tissue- and organ-level morphogenesis. Such descriptions can be linked to the underlying gene regulatory networks through the identification of transcriptionally regulated cellular effectors of cell behaviour, as pioneered during heart development [53]. The genetically and morphologically simple ascidian embryos, like those of the nematode *Caenorhabditis elegans* [87,89], are thus ideally suited to understand how static DNA sequence information is translated into the dynamic properties of cells, tissues and organs. Lack of cell growth, limited cell migration or intercalation, and the stereotyped development based on cell lineages also favour the mechanical modelling of morphogenesis [84].

In addition to sexual reproduction, colonial ascidians can also reproduce asexually via stem-cell-mediated budding, without passing through the autapomorphic chordate tadpole larval form [90]. The extent of similarity between the sexual and asexual developmental programmes, which give rise to the same adult form, is a fascinating issue that has only recently started to be explored [91–93]. Ascidians are also important in the field of innate immunity. Some colonial ascidians, including the stolidobranch ascidian *B. schlosseri*, have developed a sophisticated histocompatibility system, which determines whether two colonies will fuse or reject each other upon contact [93]. Availability of the *B. schlosseri* genome opens the way for a systematic molecular analysis of both asexual development (e.g. [94]) and allorecognition [95].

The understanding of the genomics and development of non-ascidian tunicates is also progressing. The cell lineage of the pelagic appendicularian *O. dioica* was recently described and shown to markedly differ from ascidian cell lineages [29]. Its genome has also been sequenced, revealing an astonishing genomic compaction with extensive gene losses [61,96]. Although functional interference with gene activity has been reported in *O. dioica* [97], scarce information about the genetic developmental programme of this clade is currently available. Finally, thaliaceans lag behind all tunicates. Their embryology remains very poorly understood [23] and thaliacean genomic resources are for the time being limited to a single doliolid mitochondrial genome [98].

## 7. Tunicates as models of animal evolution and developmental systems drift

The renaissance of tunicate studies at the end of the twentieth century mostly found its roots in the belief that they could shed light on the evolutionary emergence of medically relevant vertebrate processes [52,99]. We argue below that tunicate embryos are worth studying in their own right, independently of their evolutionary proximity to vertebrates, as they may provide some original and crucial insights into the process of animal evolution.

Since Darwin, the crucial role of natural selection in shaping the evolution of species has been confirmed by experimental evolution studies and by the detection of multiple signatures of selection in genome-scale data [100]. The past century has however seen successive refinements of the theory of Darwin [101]. One longstanding question is whether evolution proceeds gradually at a slow and constant rate, or whether ‘Nature does make jumps now and then’ as Huxley wondered [102]. On the basis of the absence of intermediate states in the palaeontological record, Eldredge and Gould proposed that rather than being gradual, evolution proceeds by the alternation of long phases of evolutionary stasis with brief episodes of rapid changes at the time of speciation [103]. Indeed, some lineages have kept virtually unchanged adult morphologies for tens to hundreds million years, such as the coelacanth [104] or the horseshoe crab [105], while others show very rapid morphological changes, such as the crater lake cichlids [106]. Also, while naturally selected single mutations in wild populations were predicted to have only small phenotypic effects, some with much large morphological effects have been identified [107,108]. Taken together, these observations support that evolution may, at least in some groups, proceed by periods of stasis followed by relatively rapid evolutionary change, a process coined punctuated equilibria.

As described above, solitary ascidian embryos provide an example of exceptional morphological stasis. The embryonic cell lineages of the phlebobranch *C. intestinalis* and of the stolidobranch *H. roretzi*, two species probably separated by several hundred million years of evolution, are nearly identical [109]. Even the shape, position and surface of contacts between embryonic cells appear well conserved [81,84]. Yet, profound changes in this exceptionally conserved embryogenetic programme are observed and correlate with major ecological transitions within tunicates, from sessility to pelagy as seen in thaliaceans, or from solitary to colonial lifestyle as observed in ascidians. This pattern of evolution agrees with Eldredge and Gould's theory of punctuated equilibria.

Morphological stasis occurs in a general context of the accumulation of genetic mutations in successive generations. Intuitively, one would expect the rates of morphological and genome evolution to be coupled. Indeed, coelacanth protein-coding genes are slowly evolving [110], whereas crater lake cichlid fish genomes show accelerated evolution [111]. The situation, however, appears radically different in tunicates, which show rapid evolution of protein-coding genes throughout the clade, as well as extensive divergence in non-coding sequences [96]. In addition, ascidians are among the most polymorphic animal species. The evolutionary stasis observed in solitary ascidians is thus paradoxically associated to rapid intra-specific polymorphism and interspecific genome divergence. Ascidians thus constitute an ideal system to study how genetic variability can be buffered to produce very similar embryos.

This buffering could take place at different levels. Preliminary evidence on very few proteins suggests that some proteins appear to retain their function between distantly related ascidians, while others do not [66]. In parallel, emerging evidence points to a substantial variability of gene expression patterns across tunicates [112], even between individuals of a single species [113], suggesting quantitative or qualitative changes in the gene regulatory networks underlying gene expression. To better understand the evolution of gene regulatory networks within solitary ascidians, several studies tested conservation of the function of non-coding sequences between ascidians. In most cases, the *cis*-regulatory sequences that have been tested in cross genus transgenic experiments show functional conservation in spite of a level of sequence divergence that precludes their alignment [114,115]. In such cases, upstream regulatory transcription factors (TFs) are conserved, and phenotypically cryptic sequence divergence reflects a high tolerance to TF-binding site turnover, without change in the underlying regulatory logic. Buffering of non-coding sequence divergence can thus result from the relaxed syntax of developmental *cis*-regulatory sequences, as described in other systems [116,117]. There are however also documented cases of changes in the *cis*-regulatory logic between *Molgula* and *Ciona* species, without qualitative changes in the expression profiles of the genes [66]. At least one key *Molgula* developmental gene, the zinc-finger gene *Manx* [38], has no identifiable orthologue in *C. intestinalis*. Additional cases of rewiring between stolidobranchs and phlebobranchs have been described in the notochord, and muscle lineages [65,118]. Thus, ascidian embryonic gene regulatory networks can undergo profound rewiring without obvious phenotypic consequences, a process referred to as DSD for developmental systems drift [119] or divergence [120].

The studies described in the above paragraph are still in their infancy, and only cover an anecdotic number of loci in few species. Understanding the relative contributions of protein evolution, relaxed *cis*-regulatory syntax and network rewiring to mutational robustness will require the reconstruction of homologous networks in several tunicate species, using the *Ciona* network as a guide. This will be facilitated by ongoing efforts to increase the number of tunicate species with sequenced genomes, to cover the major branches of the tunicate phylogeny. The genomes of *Phallusia mammillata* and *P. fumigata*, *H. roretzi* and *H. aurantium* are now assembled (P. L. and C. Dantec 2015, unpublished data), while drafts of the *M. oculata*, *M. occulta and M. occidentalis* genomes have recently been released [66].

What could drive the contrasted evolutionary trajectories of tunicates? Identification of genes undergoing accelerated evolution in distinct lineages may provide some insight. In addition, the high level of polymorphism in tunicates [96], besides facilitating the cloning of mutants affecting embryonic development [121], makes these animals particularly suitable to population genomics approaches [122]. This type of approach may identify signatures of natural selection on the genes or *cis*-regulatory sequences that constitute gene regulatory networks in the various clades of solitary ascidians. Indeed, a study in *C. intestinalis* revealed a high level of amino acids under either purifying or adaptive (positive) selection [123]. Comparison of patterns of selection between clades may help explain why different tunicate groups show distinct preferential evolutionary trajectories. For instance, larval tail loss has occurred independently several times in the molgulids [124], but not in other groups. Conversely, molgulids are all solitary, while aplousobranchs are all colonial and the other groups show a repeated emergence of coloniality. Population genomics may also help understand an ongoing cryptic speciation event within *C. intestinalis* [125–127].

## 8. Concluding remarks

Since Thomas Huxley, the focus of tunicate biology has been very productively displaced towards sessile ascidians, as clearly follows from the preceding discussion. The simple embryos of solitary ascidians, the contrasted evolutionary trajectories of tunicates, their richness of life histories and their high rate of polymorphism all concur to make this clade particularly attractive to study. Pelagic tunicates remain understudied, and still have many of their mysteries to give away, in particular concerning the mechanisms of adaptation to a new pelagic lifestyle. Modern imaging and molecular biology techniques should now be used to resolve some of the lasting enigmas of these fascinating animals ([23]).

In these times of harsh scientific competition, it is comforting to end with this citation of Huxley concerning Mertens' discovery of the house of appendicularians, an observation he could not confirm: ‘At the same time it is quite impossible to imagine, that an account so elaborate and detailed, can be otherwise than fundamentally true, and therefore, as Mertens' paper is not very accessible, I will add his account of the matter, trusting that further researchers may clear up the point’ [2, p. 598]. A remarkable example of scientific collegiality and ethics.
